# Blood PCSK9 Impacts Alzheimer's Disease Risk in an *APOE* Genotype‐Dependent Manner: A Prospective Cohort Study

**DOI:** 10.1002/hsr2.71810

**Published:** 2026-02-22

**Authors:** Qiushan Tao, Ting Fang Alvin Ang, Jinghan Huang, Indira Swetha Itchapurapu, Jesse Mez, Michael Alosco, Rhoda Au, Lindsay A. Farrer, Xiaoling Zhang, Wei Qiao Qiu, for the Alzheimer's Disease Neuroimaging Initiative

**Affiliations:** ^1^ Department of Pharmacology, Physiology & Biophysics Boston University Chobanian & Avedisian School of Medicine Boston Massachusetts USA; ^2^ Department of Anatomy & Neurobiology Boston University Chobanian & Avedisian School of Medicine Boston Massachusetts USA; ^3^ Slone Epidemiology Center, School of Public Health Boston University Medical Campus (BUMC) Boston Massachusetts USA; ^4^ Framingham Heart Study Boston University Chobanian & Avedisian School of Medicine Boston Massachusetts USA; ^5^ Department of Neurology Boston University Chobanian & Avedisian School of Medicine Boston Massachusetts USA; ^6^ Alzheimer's Disease and CTE Centers, Boston University Chobanian & Avedisian School of Medicine Boston Massachusetts USA; ^7^ Department of Medicine Boston University Chobanian & Avedisian School of Medicine Boston Massachusetts USA; ^8^ Department of Biostatistics Boston University School of Public Health Boston Massachusetts USA; ^9^ Department of Psychiatry Boston University Chobanian & Avedisian School of Medicine Boston Massachusetts USA

## Abstract

**Background and Aims:**

Apolipoprotein E (APOE) and proprotein convertase subtilisin/kexin type 9 (PCSK9) are both lipid proteins and related to immunity/inflammation. We hypothesized that PCSK9 impacts on Alzheimer's disease (AD) risk in an *APOE* genotype dependent manner.

**Methods:**

We used the Framingham Heart Study (FHS) Offspring cohort (Gen 2), with data on plasma PCSK9 protein concentration, as the baseline exposure for 1,704 study subjects. Using Cox regression models, the outcomes were incidents of AD or all‐cause dementia. Using another FHS dataset with 3,048 individuals with genetic data, we examined the association between PCSK9 genotypes and the incidence of AD/dementia, stratifying the analysis based on *APOE* ε4 status. The Alzheimer's Disease Neuroimaging Initiative (ADNI) study was used to validate some of the main findings.

**Results:**

Higher plasma PCSK9 protein levels were associated with a lower risk of AD (HR [95%CI]: 0.74 [0.58, 0.94]; *p* = 0.01) in *APOE* ε4 noncarriers; in contrast, PCSK9 levels were not significantly associated with AD risk in *APOE* ε4 carriers, after adjusting for common confounders, lipid profile, and lipid treatment. Using the three SNPs (rs502576, rs529787, rs676297) of the PCSK9 gene associated with PCSK9 levels in blood, we consistently found that the genotypes, which determine a low concentration of PCSK9, were associated with AD risk only in *APOE* ε4 noncarriers. These findings were validated by the ADNI study, which showed that the PCSK9 genotypes were associated with AD risk and with the AD biomarker—a low concentration of Aβ42 in CSF, only in APOE ε4 noncarriers.

**Conclusions:**

Our study suggests that high blood PCSK9 levels are protective against AD risk in *APOE* ε4 noncarriers, potentially through mechanisms related to lipid metabolism. The findings may highlight the importance of considering *APOE* genotype when prescribing the drugs targeting PCSK9.

## Summary

Both apolipoprotein E (APOE) and proprotein convertase subtilisin/kexin type 9 (PCSK9) are involved in lipid metabolism. While PCSK9 inhibitors are commonly used to treat cardiovascular disease (CVD), the potential impact of PCSK9 on Alzheimer's disease (AD) risk, particularly in the context of APOE genotype, remains unclear. Our findings suggest that low plasma levels of PCSK9 and genetic variants associated with reduced PCSK9 expression are linked to an increased risk of AD, but only in individuals *without* the APOE ε4 allele. This association was not observed in APOE ε4 carriers. Although genotypes associated with low PCSK9 levels are relatively uncommon, clinicians may need to exercise caution when prescribing PCSK9 inhibitors to hyperlipidemic patients who are APOE ε4 noncarriers and have naturally low PCSK9 levels. In such cases, alternative lipid‐lowering therapies might be considered to avoid potentially increasing AD risk.

- Why does this paper matter?
  ∘ As population ages, multiple systemetic diseases like hyperlipidemia, CVD, and AD often co‐exist in the older persons. However, it is largely understudied on how these geriatric diseases and their treatments impact on each other. PCSK9 inhibitors are a class of antilipidemic medications used for CVD. Our findings offer insights into the interactive effects of *APOE* genotypes and PCSK9 for AD. Since those patients who are *APOE* ɛ4 noncarriers and at the same time have a low level of blood PCSK9 may have a high AD risk, other antilipidemic medications, instead of those targeting PCSK9, should be used to treat hyperlipidemia for the patients.

## 1 Introduction

Alzheimer's disease (AD) is the most common neurodegenerative disorder in late life, and its etiology is considered to involve both genetic susceptibility and environmental exposures. Among genetic factors, the apolipoprotein E (APOE) gene has been most consistently implicated. Multiple cohort studies have demonstrated that the APOE ε4 allele is a major genetic risk factor for AD, while the three common isoforms (APOE2, APOE3, and APOE4) influence both amyloid‐β–dependent and independent pathways implicated in AD pathogenesis [1, 2]. Although *APOE* is primarily a lipid regulating protein, our recent studies have shown that several proinflammatory proteins including C‐reactive protein (CRP) and MCP‐1 interact with *APOE* ε4 versus *APOE* ε2 in an opposite direction to impact AD risk [3, 4]. These findings suggest that *APOE* genotypes regulate both lipid metabolism and chronic inflammation differently to mediate AD risk. Together, these observations suggest a broader role of APOE in lipid and immune pathways, prompting the hypothesis that APOE may also be linked to proprotein convertase subtilisin/kexin type 9 (PCSK9), a lipid‐protein involved in lipid metabolism via the low density lipoprotein receptor (LDLR), the very low density lipoprotein receptor (VLDLR) and apolipoprotein E receptor 2 (ApoER2) [5]. This hypothesis is further underscored by evidence that PCSK9 is also related to chronic inflammation, leading to the migration of immune cells through endothelia [6]. Recent studies have shown that cerebrospinal fluid PCSK9 levels are elevated in AD patients and correlate with APOE4 presence, indicating that ApoE4 carriers may be particularly susceptible to PCSK9‐mediated lipid dysregulation [7, 8]. This interaction could exacerbate amyloid‐beta accumulation, neuronal lipid imbalance, and neuroinflammation, highlighting the potential for APOE genotype to modulate responses to PCSK9‐targeted interventions. Basic research has discovered that PCSK9 is a key regulator of the AD pathological component Aβ clearance across the blood‐brain barrier (BBB) in the animal model [9]. In addition, PCSK9(‐/‐) mice showed increased levels of BACE1, the key protease in APP processing, and Aβ production and accumulation in the brain [10]. However, whether *APOE* genotypes interacting with PCSK9 for AD risk are unclear and understudied.

Since APOE protein regulates brain endothelia of BBB during peripheral inflammation [11], we hypothesized that blood PCSK9 impacts AD risk differently depending on the *APOE* genotypes. To address this hypothesis, we evaluated data from the Framingham Heart Study (FHS) cohort to examine the interactive effects of *APOE* genotypes and PCSK9 protein or polymorphisms for AD risk. We further used the data of the Alzheimer's Disease Neuroimaging Initiative (ADNI) study [12] to validated the findings.

## 2 Methods

This study was designed and reported in accordance with the CONSORT 2010 guidelines to ensure transparency, reproducibility, and completeness of reporting [13].

### 2.1 Participants

The first generation FHS cohort (Gen 1) was established in 1948, and the FHS Offspring Cohort started in 1971 was the second generation (Gen 2) of the original FHS cohort. Detailed information about FHS were previously reported [14]. Briefly, the FHS Offspring Cohort was strategically designed to extend and broaden the scope of the original study, delving deeper into the interplay of genetic, environmental, and lifestyle factors that influence cardiovascular health. The fifth wave of health exam (Core Exam 5) included 3799 participants (average age 55 years old, ranged 26–84) from 1991 to 1995, among which 1704 participants (age mean ± SD, 54.7 ± 9.88 years) had the data on PCSK9 protein and *APOE* genotype; in addition, 3048 participants (age mean ± SD, 54.5 ± 9.80 years) who had the genetic data on PCSK9 (SNPs genotype data from the TOPMed Project) and *APOE* genes. The exclusion citeria of the study were those missing *APOE* genotype, carrying *APOE* genotype ε2/ε4, baseline dementia when the protein was measured, and those who had follow‐up time < 3 years (Supporting Figure S1).

To validate our findings, cross‐validation analyses were performed using 1,476 participants from the Alzheimer's Disease Neuroimaging Initiative (ADNI) database (https://adni.loni.usc.edu). ADNI is a longitudinal, multicenter study designed to develop clinical, imaging, genetic, and biochemical biomarkers for the early detection and progression of Alzheimer's disease. For this analysis, logistic regression models were used to assess the association between PCSK9 genotypes and prevalent AD or all‐cause dementia, stratified by APOE ε4 carrier status. Results were reported as odds ratios (ORs) with 95% confidence intervals.

Ethical approval for this study was obtained from the Institutional Review Boards (IRBs) of Boston University Medical Center for the Framingham Heart Study (FHS) and from the respective IRBs overseeing the Alzheimer's Disease Neuroimaging Initiative (ADNI). All participants in the original cohorts provided written informed consent for study participation and data sharing. For the present secondary analysis, only deidentified and anonymized data were used, and no personally identifiable information was included. In accordance with FHS and ADNI policies and applicable IRB regulations, the use of deidentified data for secondary research purposes did not require additional informed consent.

### 2.2 PCSK9 Protein

The blood level of PCSK9 protein were measured by using the Aptamer Proteomic Profiling Lab Assay, and the detailed protocol was previusely reported [15]. In brief, the blood samples were collected in K2EDTA‐treated tubes in PMI patients and citrate‐treated tubes in FHS participants. Samples were centrifuged within 15 min at 2000g for 10 min to pellet cellular elements. The supernatant plasma was then aliquoted and frozen at −80°C. The SOMAscan™ proteomic profiling platform uses single‐stranded DNA aptamers that target 1,129 proteins. All assays were performed using SOMAscan reagents according to the manufacturer's detailed protocol [15].

### 2.3 PCSK9 SNPs

The NHLBI Trans‐Omics for Precision Medicine (TOPMed) program provided whole genome sequence (WGS), performed at several sequencing centers using DNA from blood. FHS WGS was conducted as part of TOPMed. Sequencing, performed at the Broad Institute of MIT and Harvard, achieved > ×30 depth of coverage. Details regarding the laboratory methods, data processing and quality control were previusely reported [16, 17]. Initially, all single nucleotide polymorphisms (SNPs) in the PCSK9 gene were identified. Genetic variants with minor allele frequencies of > 5% were used, and 3 top SNPs (rs502576, rs529787, and rs676297) were selected based on the association with PCSK9 protein (β > 0.20 and *p* < 0.001) in this study.

### 2.4 Demenita and AD

Dementia surveillance and diagnosis methods for FHS were previously published [3, 18]. In brief, participants with lower Mini‐Mental State Examinations (MMSE) scores were further evaluated by a comprehensive neuropsychological battery and subsequent neurological assessments. Participants flagged for possible cognitive impairment prompted by self‐ or family‐reported decline, physician referral, or review of medical records would also receive annual neurological and neuropsychological examinations.

A dementia review panel, consisting of a neurologist and a neuropsychologist, adjudicated all suspected cases. Dementia diagnoses were made according to DSM‐IV criteria, based on longitudinal cognitive assessments, caregiver interviews, medical records, neuroimaging, and autopsy data when available. Importantly, diagnoses of Alzheimer's disease dementia (ADD) were determined using NINCDS‐ADRDA criteria, thereby distinguishing ADD from preclinical AD or mild cognitive impairment (MCI).

### 2.5 Covariates

Covariates in our analyses include sex, age, education, body mass index (BMI; kg/m^2^), and medical histories gathered at the baseline. Educational attainment was categorized as no high school graduation, high school graduated, any college degrees, or > college degree. Cardiovascular diseases (CVDs) defined as incident coronary heart disease, stroke, heart failure, and peripheral arterial disease. The lipid profile, encompassing total cholesterol (TC), triglycerides (TG), HDL‐Cholesterol (HDL‐C), and calculated LDL‐Cholesterol (LDL‐C) using the Friedewald formula (LDL‐C = TC ‐ HDL‐C ‐ TG/5), was obtained from blood tests. Additionally, we computed the cumulative percentage of time on anti‐lipid medication use to gauge cumulative exposure to lipid‐lowering treatment ([number of core exams with medication use/nubmer of attended observation core examinations] × 100).

### 2.6 Statistical Analysis

Descriptive statistics were used to summarize baseline characteristics of the study population. Continuous variables were presented as means ± standard deviations or medians with interquartile ranges if the distribution was skewed. Categorical variables were summarized as frequencies and percentages (n, %). In case of missing data less than 10%, we assumed the data were missing at random and still employed complete case analysis in the multivariate analysis.

Dementia cases were defined as incident, new‐onset all‐cause dementia occurring after the baseline exam, with approximately 73% classified as AD dementia. Analyses included all participants at risk using the COX proportional hazards models to assess the association between the exposure variable (blood PCSK9 protein concentration or PCSK9 genotypes) and the follow‐up outcomes of the incident AD and all‐cause dementia. Adjusted analyses were conducted to control for potential confounders including age, sex, education, lipid profile and lipid treatments. The results were reported as hazard ratios (HR) with corresponding 95% confidence intervals (95% CI). Sensitivity analyses were performed to evaluate the robustness of our findings. The sensitivity analysis comprised stratified (subgroup) analyses and interaction testing.

Cross‐validation was performed using logistic regression model (the results were reported as odds ratio (OR) with its 95% CI) of the association between PCSK9 genotypes and prevlent of AD or all‐cause dementia among 1476 ADNI participants to validate the association between AD/Dementia and the selected SNPs of PCSK, stratified by *APOE* genotype.

All statistical analyses were conducted using R: A language and environment for statistical computing (version 4.3.1). Effect sizes are presented with 95% confidence intervals to convey both magnitude and precision. *p* values are reported following standard conventions (*p* < 0.001, to the nearest thousandth for 0.001–0.01, to the nearest hundredth for ≥ 0.01, and *p* > 0.99 where applicable). A two‐sided significance level of 0.05 was applied for all tests. All statistical analysis and reporting adhered to recommended standards for clinical research, following the Statistical Analyses and Methods in the Published Literature (SAMPL) guidelines and standard references [19].

## 3 Results

### 3.1 Characterization of the Samples Used for the Data Analyses

We used two datasets from the FHS Gen 2 cohort, exam 5, 1) the protein dataset was the one with the measurements of plasma PCSK9 protein (*n* = 1704) and 2) the genetic dataset was the one with the genetic data on PCSK9 gene (*n* = 3048) (Supporting Figure S1). Two datasets were compatible in basic characteristics including age (mean ± SD: 54.7 ± 9.88 vs. 54.5 ± 9.80 years), sex, education level, and *APOE* ɛ4 carriers (Supporting Table S1). In addition, the rate of CVD, the average BMI, lipid profiles and lipid treatment were similar between two datasets.

### 3.2 Characterization of the Relationship Between the Interactive Effects of *APOE* Genotypes and Plasma PCSK9 Protein for AD Risk

We first used the data on plasma PCSK9 from the dataset, which has the plasma protein concentrations measured by proteomics. There was no difference in plasma PCSK9 concentrations between *APOE* ɛ4 noncarriers and *APOE* ɛ4 carriers and among different *APOE* genotypes (Supporting Figure S2). Using unadjusted COX regression model (Table 1, **model 1**), we found an association between plasma PCSK9 and AD (HR [95%CI]: 0.85 [0.73, 0.99], *p* = 0.04). After adjusing for age, sex, education and *APOE* ɛ4, the association between plasma PCSK9 protein and AD risk became insignificant (Table 1, **model 2**). However, the interaction of *APOE* ɛ4 genotype and plasma PCSK9 protein was significantly associated with AD risk in the same COX regression model (HR [95%CI]: 1.59 [1.12, 2.27], *p* = 0.01, Table 1, **Model 3**). This interactive effect remained and strengthened after adding lipid profiles and lipid treatment in the COX model (HR [95%CI]: 1.80 [1.24, 2.61], *p* = 0.002, Table 1, **Model 4**). The similar interactive effects between *APOE* ɛ4 genotype and plasma PCSK9 were found for all‐cause dementia, where elevated peripheral PCSK9 protein is consistently associated with a reduced hazard of developing all‐cause dementia in the overall sample (e.g., Model 4: HR = 0.81 [0.66, 0.98], *p* = 0.033), and this association is strengthened when controlling for cardiovascular risk factors. Critically, similar to the AD findings, the protective association of higher PCSK9 is not present in APOE4 carriers; rather, in these individuals, the interaction between APOE4 and PCSK9 signifies increased risk (Model 4 interaction: HR = 1.65 [1.20, 2.28], *p* = 0.002) (Table 1). The data suggested that plasma PCSK9 impact AD dementia differently based on *APOE* ɛ4 noncarriers versus carriers, with similar patterns observed for both AD dementia and all‐cause dementia.

**Table 1:** The interaction effects between PCSK9 and APOE4 carrier status for the risk of Alzheimer's disease or all‐cause dementia.

Models	Exposure	Alzheimer's disease^a^				All‐cause dementia			
		*n*	Events	HR [95% CI]	*p* value	*n*	Events	HR [95% CI]	*p* value
**Model 1**									
	PCSK9	1655	136	0.85 [0.73, 0.99]	0.043	1704	185	0.88 [0.77, 1.01]	0.080
**Model 2**									
	PCSK9	1634	135	0.94 [0.79, 1.12]	0.471	1682	183	0.97 [0.84, 1.13]	0.707
**Model 3**									
	PCSK9	1634	135	0.80 [0.65, 0.99]	0.039	1682	183	0.85 [0.71, 1.01]	0.064
	Interaction *APOE* ε4 * PCSK9	1634	135	1.59 [1.12, 2.27]	0.010	1682	183	1.54 [1.13, 2.09]	0.006
**Model 4**									
	PCSK9	1585	127	0.71 [0.57, 0.90]	0.004	1629	171	0.81 [0.66, 0.98]	0.033
	Interaction *APOE* ε4 * PCSK9	1585	127	1.80 [1.24, 2.62]	0.002	1629	171	1.65 [1.20, 2.28]	0.002

*Note:* Using the FHS protein dataset, Cox regression analysis was conducted to examine the association between blood PCSK9 protein levels and the incidence of AD or all‐cause dementia over a 30‐year follow‐up. Hazard ratios (HR) and 95% confidence intervals (95% CI) are reported for four models: Model 1, an unadjusted univariate model; Model 2, adjusted for sex, age, education, APOE ε4 genotype (ε34, 44), and PCSK9 protein (z‐score); Model 3, further including an interaction term between APOE ε4 and PCSK9 protein; and Model 4, additionally adjusting for cardiovascular diseases (CVDs, defined as coronary heart disease, stroke, heart failure, or peripheral arterial disease), BMI, triglycerides (TG), HDL, LDL, and cumulative use of anti‐lipid medication.

^a^ When AD was the outcome, other dementia cases were excluded.

Next, we stratified the participants into *APOE* ɛ4 noncarriers and carriers. Among the noncarriers, plasma PCSK9 concentration was negative associated with AD risk after adjusting for age, sex, education, lipid profiles and lipid treatment (HR [95%CI]: 0.74 [0.58, 0.94], *p* = 0.01) (Table 2). In contrast, in *APOE* ɛ4 carriers, plasma PCSK9 concentration tended to be positively associated with AD risk but did not reach statistical significance. Similar tendencies of the associations were found for all‐cause dementia in an *APOE* genotype dependent manner (Table 2). Using the Kaplan‐Meier analyses, there was a tendency of low concentration of plasma PCSK9 increasing AD risk in in *APOE* ɛ4 noncarriers (*p* = 0.13) (Supporting Figure S3).

**Table 2:** Stratification and COX regression analyses for the association between blood PCSK9 protein level and the AD or all‐cause dementia incident up to 30 years of follow‐up in FHS in the absence and the presence of APOE ε4 genotype.

Strata	Models	Alzheimer's disease^a^				All‐cause dementia			
		*n*	Events	HR [95CI]	*p* value	*n*	Events	HR [95CI]	*p* value
**APOE4 (−)**									
	**Model 1**	1290	93	0.78 [0.63, 0.96]	0.021	1325	128	0.83 [0.69, 0.99]	0.040
	**Model 2**	1250	87	0.74 [0.58, 0.94]	0.013	1281	118	0.84 [0.69, 1.03]	0.100
**APOE4 (+)**									
	**Model 1**	344	42	1.29 [0.97, 1.73]	0.084	357	55	1.33 [1.03, 1.72]	0.030
	**Model 2**	335	40	1.22 [0.85, 1.74]	0.280	348	53	1.25 [0.92, 1.70]	0.159

*Note:* FHS participants were categorized by APOE ε4 status, with APOE4 (−) defined as non‐carriers (ε22, ε23, and ε33) and APOE4 (+) as carriers (ε34 and ε44), while APOE ε24 carriers were excluded. Cox regression analysis was performed separately for each APOE genotype, reporting hazard ratios (HR) and 95% confidence intervals (95% CI). Model 1 adjusted for sex, age, education, APOE ε4, and PCSK9 protein (z‐score), while Model 2 further adjusted for cardiovascular diseases (CVDs, defined as incident coronary heart disease, stroke, heart failure, and peripheral arterial disease), BMI, triglycerides (TG), HDL, LDL, and cumulative use of anti‐lipid medication.

^a^ When AD incidence was the outcome, other dementia diagnoses were excluded.

### 3.3 Characterization of the Relationship Between the Interactive Effects of the *APOE* and PCSK9 Genotypes for AD Risk

Next, we used the larger sample from FHS Gen 2 which has genetic information (*n* = 3048) (Supporting Figure S1 and Supporting Table S1). Among the PCSK9 polymorphisms, we chosed the top three PCSK9 gene SNPs, rs502576, rs676297 and rs529787, based on their influences on the plasma level of PCSK9 protein (β level > 0.20 in the linear regression model) (Supporting Table S2. These SNPs are all located in the 3rd intron of the PCSK9 gene (Supporting Figure S4). The 3 genotypes were significantly associated with plasma PCSK9 protein in the whole sample, especially in *APOE* ɛ4 noncarriers, but their influences on plasma PCSK9 protein in the presence of *APOE* ɛ4 were not significant (Supporting Figure S5). We combined *APOE* ɛ3/4 and *APOE* ɛ4/4 for the category of *APOE* ɛ4 because of the low numbers in *APOE* ɛ4/4 (Supporting Table S3 **)**.

**Figure 1:**
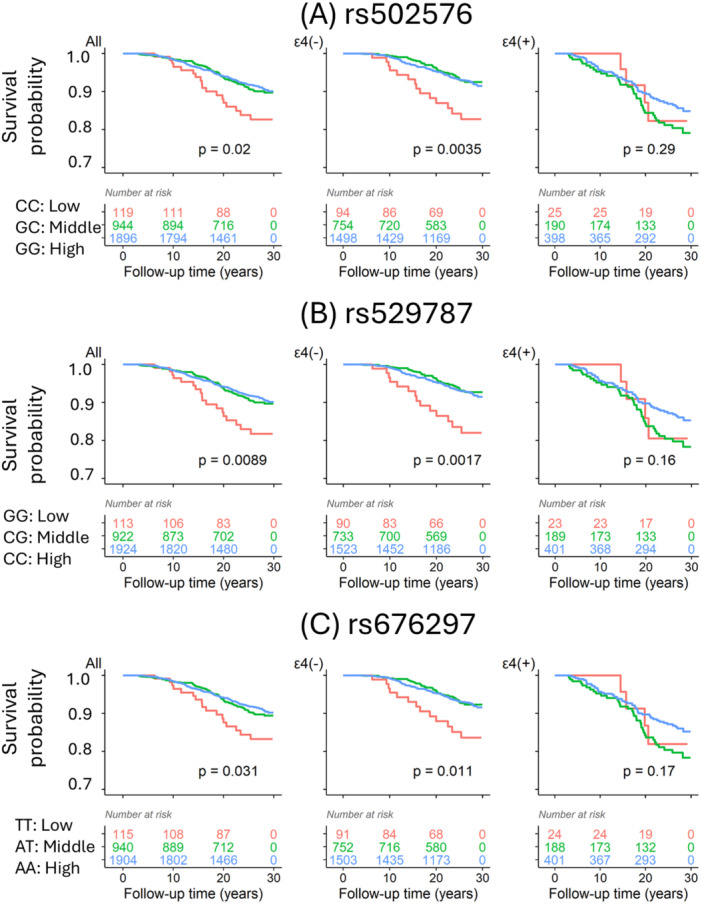
Kaplan‐Meier plot illustrating the survival analysis for Alzheimer's disease (AD) across different PCSK9 genotypes in the FHS study. The genotypes of three PCSK9 SNPs were categorized as “Low”, “Middle”, and “High” based on their association with blood PCSK9 protein levels in the APOE ε4 non‐carriers groups as shown in Supporting Figure S5, respectively. Survival analysis was conducted in the total sample (All), APOE ε4 non‐carriers (APOE4−), and APOE ε4 carriers (APOE4 +). Kaplan‐Meier curves for AD survival are shown for rs502576 (A) – CC (Low), GC (Middle), and GG (High); rs529787 (B) – GG (Low), CG (Middle), and CC (High); and rs676297 (C) – TT (Low), AT (Middle), and AA (High). Genotypes associated with lower PCSK9 protein concentrations exhibited a higher risk of AD development compared to other genotypes.

Using COX model after adjusting for age, sex, education, lipid profiles and lipid treatment, we consistently found a negative association between those PCSK9 genotypes determining a low concentration of PCSK9 protein (Supporting Figure S5) and AD risk in *APOE* ɛ4 noncarriers (Table 3), which showed a similar trend of plasma PCSK9 protein and AD risk. Again these relationships were not found in *APOE* ɛ4 carriers. Using Kaplan‐Meier analyses, those genotypes determining a low concentration of PCSK9 protein in plasma were associated with a high risk of AD onset in the absence of *APOE* ɛ4 (Figure 1), but there were no associations between these SNPs and AD risk among those *APOE* ɛ4 carriers.

**Table 3:** Stratification and COX regression analyses for the association between PCSK9 genotypes and the AD or all‐cause dementia up to 30 years of follow‐up in FHS in the absence and the presence of APOE ε4 genotype.

Strata	SNP	Genotype	Alzheimer's disease				All‐cause dementia			
			*n*	Events	HR [95CI]	*p* value	*n*	Events	HR [95CI]	*p* value
**APOE4 (−)**	**rs502576**	CC: Low	94	14	ref	—	96	16		
		GC: Middle	740	46	0.28 [0.15, 0.52]	< 0.001	762	68	0.40 [0.23, 0.69]	0.001
		GG: High	1482	99	0.30 [0.17, 0.53]	< 0.001	1523	140	0.41 [0.24, 0.70]	< 0.001
	**rs529787**	GG: Low	90	14	ref	—	92	16	ref	—
		CG: Middle	733	44	0.26 [0.14, 0.49]	< 0.001	755	66	0.38 [0.22, 0.66]	< 0.001
		CC: High	1523	101	0.29 [0.17, 0.51]	< 0.001	1566	144	0.40 [0.24, 0.67]	< 0.001
	**rs676297**	TT: Low	91	13	ref	—	93	15	ref	—
		AT: Middle	739	47	0.30 [0.16, 0.56]	< 0.001	758	66	0.41 [0.23, 0.72]	0.002
		AA: High	1486	99	0.32 [0.18, 0.57]	< 0.001	1530	143	0.44 [0.25, 0.75]	0.003
**APOE4 (+)**	**rs502576**	CC: Low	25	4	ref	—	26	5	ref	—
		GC: Middle	186	33	1.62 [0.57, 4.63]	0.37	195	42	1.52 [0.60, 3.89]	0.38
		GG: High	395	51	1.36 [0.48, 3.84]	0.56	406	62	1.26 [0.50, 3.20]	0.62
	**rs529787**	GG: Low	23	4	ref	—	24	5	ref	—
		CG: Middle	189	35	1.63 [0.57, 4.65]	0.36	199	45	1.52 [0.60, 3.87]	0.38
		CC: High	401	50	1.34 [0.48, 3.79]	0.78	412	61	1.25 [0.49, 3.16]	0.64
	**rs676297**	TT: Low	24	4	ref	—	25	5	ref	—
		AT: Middle	184	34	1.25 [0.44, 3.56]	0.68	193	43	1.10 [0.43, 2.81]	0.84
		AA: High	398	50	1.05 [0.37, 2.95]	0.92	409	61	0.93 [0.37, 2.33]	0.87

*Note:* FHS participants at exam 5 were classified by APOE ε4 status: APOE4 (−) (ε22, ε23, ε33) and APOE4 (+) (ε34, ε44), excluding ε24 carriers. Genotypes were categorized as rs502576 (CC‐Low, GC‐Middle, GG‐High), rs529787 (GG‐Low, CG‐Middle, GG‐High), and rs676297 (TT‐Low, AT‐Middle, AA‐High). The label “Low”, “Middle”, and “High” of each genotypes were based on the PCSK protein levels in the APOE ε4 non‐carriers group as shown in the Supporting Figure 5, respectively. Using the lowest PCSK9 genotype as reference, Cox regression estimated hazard ratios (HR) and 95% confidence intervals (CI) for AD or all‐cause dementia within each APOE group.

### 3.4 Validation of the Relationship Between the Interactive Effects of the *APOE* and PCSK9 Genotypes for AD Risk in the ADNI Study

We used the ADNI dataset and logistic model to validate the findings in FHS. After adjusting for age, sex and education, three PCSK9 polymorphisms, rs502576, rs676297, and rs529787, in the ADNI study were found to replicate the results in FHS (Table 3), e.g the PCSK9 SNPs were associated with AD dementia in *APOE* ɛ4 noncarriers, but not in *APOE* ɛ4 carriers (Supporting Table S4).

**Figure 2:**
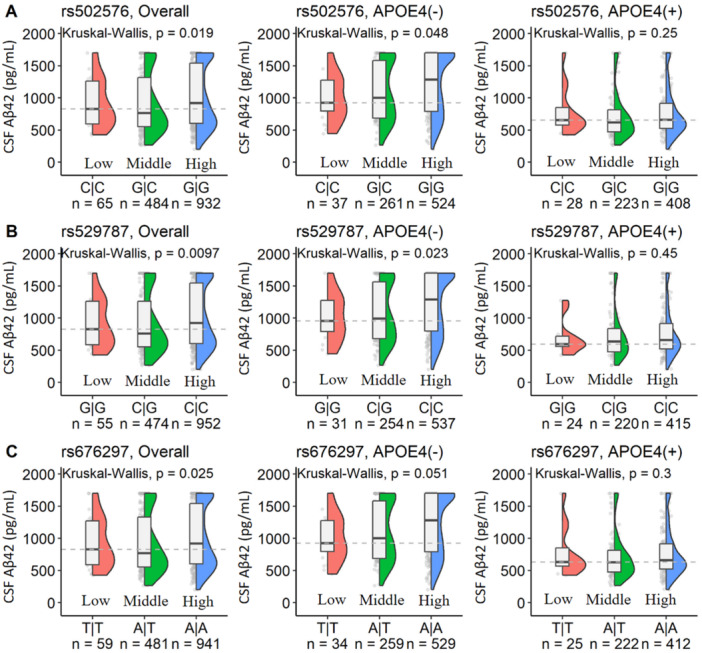
The boxplots for the CSF Aβ42 levels across the genotypes of PCSK9 gene stratified by *APOE* ε4 status in the ADNI study. Three SNPs in the PCSK9 gene were analyzed, with genotypes labeled as “Low”, “Middle”, and “High” based on corresponding blood PCSK9 protein levels in the APOE ε4 non‐carriers groups as shown in Supporting Figure S5, respectively. The genotypes were defined as follows: rs502576 (A) – CC (Low), GC (Middle), and GG (High); rs529787 (B) – GG (Low), CG (Middle), and CC (High); and rs676297 (C) – TT (Low), AT (Middle), and AA (High). CSF Aβ42 levels were compared across these genotype groups in the total sample (All), in APOE ε4 non‐carriers (APOE4−), and in APOE ε4 carriers (APOE4 +). The reported p‐values were from the non‐parametric Kruskal‐Wallis tests.

Since the basic research study discovered that PCSK9 acts as a key regulator of Aβ clearance [9] and the ADNI dataset has the data on the AD biomarkers in CSF, we examined the associations between the interactive effects of *APOE* ɛ4 and PCSK9 genotypes for the AD biomarkers in CSF. After adjusting for age, sex and education, we found that all three PCSK9 polymorphisms rs502576, rs676297, and rs529787, for a low concentrations of plasma PCSK9 had a lower level of CSF Aβ42, a biomarker indicating AD diagnosis, in *APOE* ɛ4 noncarriers, but not in *APOE* ɛ4 carriers (Figure 2). However, we did not find the differences of CSF total Tau (Supporting Figure S6) and pTau (Supporting Figure S7) across 3 PCSK9 genotypes of all three SNPs regardless of *APOE* ɛ4.

## 4 Discussion

Our study demonstrated a genotype‐specific relationship between PCSK9 and AD risk. Among *APOE ε4* noncarriers, higher plasma PCSK9 levels were associated with a 26% (HR [95% CI]: 0.74 [0.58, 0.94]) lower risk of AD, and this protective association was consistent across both protein and genetic analyses. In contrast, among *APOE ε4* carriers, neither plasma PCSK9 levels nor PCSK9 genotypes were significantly associated with AD risk (Table 2), indicating that the protective effect of PCSK9 is limited to noncarriers. Clinically, these findings suggest that noncarriers with higher PCSK9 may have substantially reduced AD risk, whereas ε4 carriers do not appear to benefit. Moreover, the beneficial association of higher circulating PCSK9 may extend beyond AD to reduce the risk of all‐cause dementia. The genotype‐dependent nature of this protective effect—restricted to non‐APOE ε4 carriers—likely reflects fundamental interactions between cholesterol/lipid pathways and APOE ε4–mediated neurodegeneration. The parallel risk patterns observed for both AD and all‐cause dementia reinforce the potential generalizability of PCSK9's neuroprotective role and highlight the importance of considering APOE ε4 status when evaluating therapeutic strategies or biomarker utility, particularly given the broad clinical and societal impact of all‐cause dementia.

As some proinflammatory proteins in blood impact *APOE* genotypes differently for AD risk [4, 20], PCSK9, a lipid related protein, also showed different or opposite associations with AD risk depending on *APOE* genotypes, e.g blood PCSK9 could be a protector for AD in *APOE* ɛ4 noncarriers but did not influence AD risk in *APOE* ɛ4 carriers. Consistent with our findings, other studies show the potential risks that may arise from low PCSK9 level (variant or inhibitor) for central nervous system (CNS) diseases including AD [21]. In the absence of *APOE* ɛ4 in humans, the genotypes associated with low concentrations of blood PCSK9 had a low level of Aβ42 in CSF, a diagnostic biomarker of AD, but this relationship did not appear among *APOE* ɛ4 carriers (Figure 2). This is consistent with the preclinical study that PCSK9(‐/‐) knockout mice showed increased levels of BACE1, leading to increased Aβ production and accumulation in the brain [10]. However, some studies investigate the central PCSK9 effects in the brain by using the AD mouse models with Aβ deposits in the brain with conflict results. For example, it was found that increased the brain expression of PCSK9 induced atherosclerosis, brain hypoxia and Aβ deposits in the brain [22]. In addition, knock‐down the expression of PCSK9 in the brain decreases Aβ level and neuroinflammation, and improve behavioral tests in these mice [23]. As our study focused on the effects of peripheral, but not central, PCSK9 for AD, the mechanism of peripheral PCSK9 for the brain is unclear. It is reported that PCSK9 plays a role in neurogenesis, neural cell differentiation, central LDL receptor metabolism, neural cell apoptosis, neuroinflammation, AD, alcohol use disorder, and stroke [24] that may explain the beneficial effects of peripheral PCSK9 on AD risk in the absence of *APOE* ɛ4.

The interactive effects with *APOE* ɛ4 noncarrier genotypes and increased plasma PCSK9 for reduced AD risk were influenced by adding lipid profile and anti‐lipid medications into the models (Tables 1 and 2). As some lipid species are beneficial for brain functions [25], the beneficial effects of peripheral PCSK9 on AD pathogenesis were probably through regulating lipid metabolism. Thus, diet components and taking the drugs targeting peripheral lipid metabolism could influence the associations between PCSK9 and AD. As taking the statin medications become common among midlife and old age in recent decades, lipid profiles in different populations have been changed and improved. The FHS cohort in this study only had < 5% of participants taking anti‐lipid medications in the 1990s, while the ADNI study was in early and the UKBB study was in late decade of 2000. A study using the UK Biobank (UKBB) cohort did not find that PCSK9, either protein or genotypes, is associated with AD risk [26].

While three genotypes were associated with blood PCSK9 concentrations in the absence of *APOE* ɛ4, the PCSK9 genotypes did not link with PCSK9 protein levels in the presence of *APOE* ɛ4 (Supporting Figure S5). APOE4 protein may suppress the expression of PCSK9 to inhibit PCSK9 regulating the pathways of immune responses and lipid metabolism. There was a trend of positive association between PCSK9 and AD risk in the presence of APOE4 (Table 2) but did not reach statistical significance (Tables 2 and 3; Figure 1) probably due to the lack of power. As lowering cholesterol in APOE4 carriers may decrease the risk of dementia based on other published literature [27, 28, 29, 30, 31, 32], it is necessary and probably safe for clinicians to treat hyperlipidemic patients who are APOE ε4 carriers with anti‐lipid medications including PCSK9 inhibitors.

PCSK9 inhibitors are the effective drugs to reduce the cholesterol levels and atherosclerosis to decrease the risk of cardiovascular diseases [5], but both cohorts we used did not have the data on PCSK9 inhibitor use. Up to date, the conclusions on PCSK9 inhibitors and AD risk are not conclusive. For example, in some trials, PCSK9 inhibitors including evolocumab do not show the effects on cognitive decline and complain in a large clinical trial [33, 34]. PCSK9 inhibitors may be harmful for AD in the minor frequencies of some PCSK9 genotypes [21, 24]. Another study did not show the interactive effects with *APOE* ɛ4 genotype for cognitive decline and complain in a large clinical trial [33]. In basic research, peripheral injection of PCSK9 inhibitors into the AD mouse models are shown to decrease Aβ level and neuroinflammation in the brain, and improve behavioral tests [9, 35].

While multiple factors including other genes, diets and anti‐lipid medication use can affect lipid metabolism and AD risk [36], our study did not account for diet as a potential confounding factor. Another limitation was the absence of experimental validation; however, future basic research is needed to investigate the mechanisms underlying the interaction between PCSK9 and APOE genotype in influencing AD risk. A third limitation was the lack of longitudinal PCSK9 measurements, which prevents drawing causal inferences regarding AD risk. Additionally, the lack of ethnic diversity of the FHS cohort may restrict the generalizability of our findings to more diverse populations. Note that residual confounding by age remains a potential limitation, as our primary analyses did not use age‐matched sampling. Future studies employing age‐matched replication analyses would help to confirm the robustness of these findings. A limitation of our study is that corrections for multiple comparisons were not applied, which may increase the risk of type I error. Nonetheless, the analyses were hypothesis‐driven and focused on predefined outcomes, which mitigates (but does not eliminate) this concern. Despite these limitations, the observed interaction between APOE genotypes and PCSK9 in our study may help explain some of the inconsistencies reported across different cohorts and clinical trials involving PCSK9 inhibitors in the context of AD. Clinical practice may need to avoid prescribing the PCSK9 inhibitors, but other anti‐lipid medications, for cardiovascular patients who are *APOE* ɛ4 noncarriers and carry the genotypes for low blood PCSK9 to reduce AD risk.

## Author Contributions

**Qiushan Tao:** conceptualization, data curation, formal analysis, methodology, writing – original draft, writing – review and editing. **Ting Fang Alvin Ang:** conceptualization, data curation, formal analysis, methodology, supervision. **Jinghan Huang:** methodology. **Indira Swetha Itchapurapu:** project administration, Resources. **Jesse Mez:** writing – review and editing. **Michael Alosco:** writing – review and editing. **Rhoda Au:** conceptualization, funding acquisition, supervision, writing – review and editing. **Lindsay A. Farrer:** conceptualization, funding acquisition, supervision, writing – review and editing. **Xiaoling Zhang:** methodology, writing – review and editing. **Wei Qiao Qiu:** conceptualization, investigation, methodology, project administration, supervision, writing – original draft, writing – review and editing.

## Ethics Statement

Ethical approval for this study was obtained through the respective Institutional Review Boards (IRBs) overseeing the Alzheimer's Disease Neuroimaging Initiative (ADNI) and the Framingham Heart Study (FHS). All human subjects in the original studies provided informed consent for participation and data sharing. For this secondary analysis, only deidentified, anonymized data were used, and no personally identifiable information (PII) was included. According to the policies of ADNI and FHS and in adherence with applicable IRB regulations, the use of such deidentified data for secondary research purposes did not require additional informed consent from participants.

## Conflicts of Interest

The authors declare no conflicts of interest.

## Transparency Statement

The lead author Wei Qiao Qiu affirms that this manuscript is an honest, accurate, and transparent account of the study being reported; that no important aspects of the study have been omitted; and that any discrepancies from the study as planned (and, if relevant, registered) have been explained.

## Supporting information


**Supplemental Figure S1:** The flow‐chart of inclusion and exclusion of the study participates. **Supplemental Figure S2:** The violin‐boxplot shows the plasma PCSK9 concentrations between the two groups, APOE4(+) (APOE ɛ4 noncarriers) versus APOE4(+) (APOE ɛ4 carriers) (A); among different APOE genotypes (B). **Supplemental Figure S3:** Kaplan‐Meier Plot Illustrating the Survival Analysis for Alzheimer's Disease (AD) in Relation to PCSK9 Protein Levels. **Supplemental Figure S4:** The LocusZoom plot of the three selected SNPs and their association with PCSK9 protein levels. **Supplemental Figure S5:** The boxplots of the blood PCSK9 protein levels across three PCSK9 genotypes in the absence and the presence of APOE ε4 allele. **Supplemental Figure S6:** The boxplots of the CSF total Tau levels among different genotypes of the three PCSK9 SNPs in the ADNI study at the baseline exams, stratified by APOE ε4 carriers' status. **Supplemental Figure S7:** The boxplot shows the CSF pTau levels among different genotypes of the three PCSK9 SNPs in the ADNI study at the baseline exams, stratified by APOE ε4 carriers' status. **Supplemental Table S1:** Baseline characteristics of two FHS datasets: the protein dataset and the genetic dataset. **Supplemental Table S2:** The association between PCSK9 protein level and the dosage of PCSK9 SNPs. **Supplemental Table S3:** The numbers of study subjects in the stratification of PCSK9 genotypes in the absence and the presence of APOE ε4 genotype. **Supplemental Table S4:** Stratification and logistic regression analyses for the association between PCSK9 genotypes and the AD or all‐cause dementia in ADNI study in the absence and the presence of APOE ε4 genotype.

## Data Availability

The data (FHS and ADNI) used in this study are not publicly shared by the authors. However, they are available upon request from the Framingham Heart Study (FHS) through a formal data request process (https://www.framinghamheartstudy.org/fhs-for-researchers/research-application/) and ADNI (https://adni.loni.usc.edu/data-samples/adni-data/#AccessData). Researchers may access the data by submitting a research proposal to the FHS and ADNI, subject to review and approval in accordance with their data access policies.
