# Supplementary material for: Blood PCSK9 Impacts Alzheimer's Disease Risk in an APOE Genotype‐Dependent Manner: A Prospective Cohort Study
Source: Health Sci Rep. 2026 Feb 22;9(2):e71810. doi: 10.1002/hsr2.71810 (PMC12927996; doi:10.1002/hsr2.71810)
Supplement: Supplementary file 1 — Supplemental Figure S1: The flow‐chart of inclusion and exclusion of the study participates. Supplemental Figure S2: The violin‐boxplot shows the plasma PCSK9 concentrations between the two groups, APOE4(+) (APOE ɛ4 noncarriers) versus APOE4(+) (APOE ɛ4 carriers) (A); among different APOE genotypes (B). Supplemental Figure S3: Kaplan‐Meier Plot Illustrating the Survival Analysis for Alzheimer's Disease (AD) in Relation to PCSK9 Protein Levels. Supplemental Figure S4: The LocusZoom plot of the three selected SNPs and their association with PCSK9 protein levels. Supplemental Figure S5: The boxplots of the blood PCSK9 protein levels across three PCSK9 genotypes in the absence and the presence of APOE ε4 allele. Supplemental Figure S6: The boxplots of the CSF total Tau levels among different genotypes of the three PCSK9 SNPs in the ADNI study at the baseline exams, stratified by APOE ε4 carriers' status. Supplemental Figure S7: The boxplot shows the CSF pTau levels among different genotypes of the three PCSK9 SNPs in the ADNI study at the baseline exams, stratified by APOE ε4 carriers' status. Supplemental Table S1: Baseline characteristics of two FHS datasets: the protein dataset and the genetic dataset. Supplemental Table S2: The association between PCSK9 protein level and the dosage of PCSK9 SNPs. Supplemental Table S3: The numbers of study subjects in the stratification of PCSK9 genotypes in the absence and the presence of APOE ε4 genotype. Supplemental Table S4: Stratification and logistic regression analyses for the association between PCSK9 genotypes and the AD or all‐cause dementia in ADNI study in the absence and the presence of APOE ε4 genotype. [file HSR2-9-e71810-s001.pdf]

Supplemental Tables and Figures

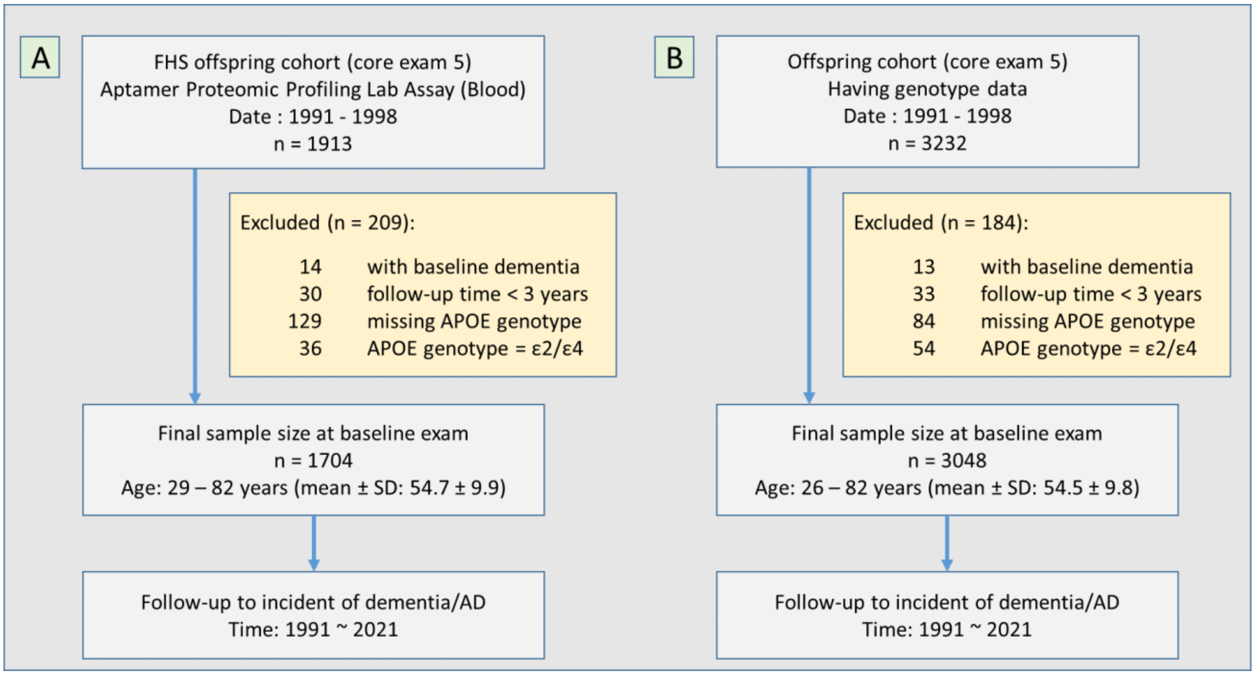

Supplemental Figure S1. The flow-chart of inclusion and exclusion of the study participates

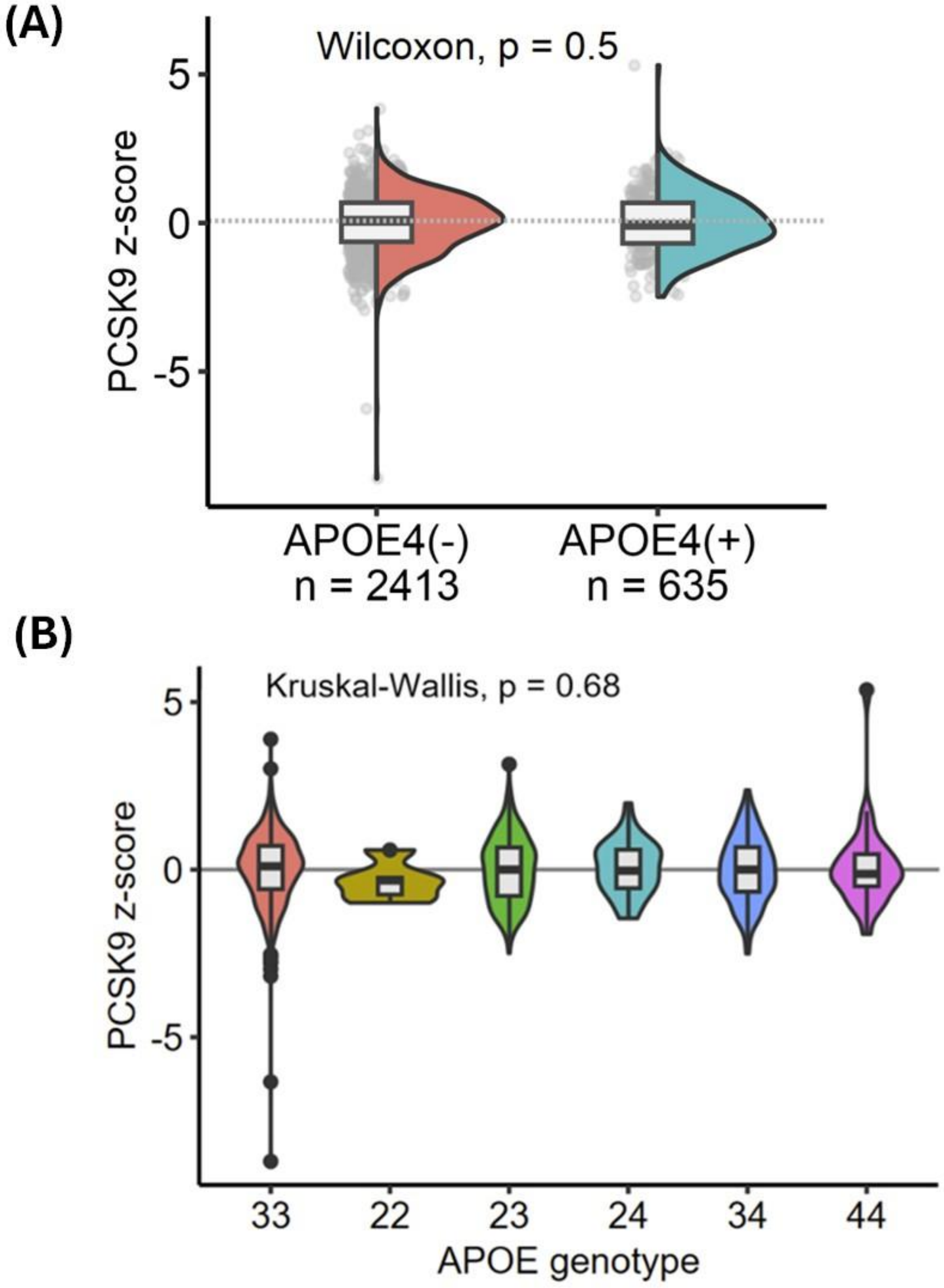

Supplemental Figure S2. The violin-boxplot shows the plasma PCSK9 concentrations between the two groups, APOE4(+) (*APOE* ε4 noncarriers) versus APOE4(+) (*APOE* ε4 carriers) (A); among different *APOE* genotypes (B).

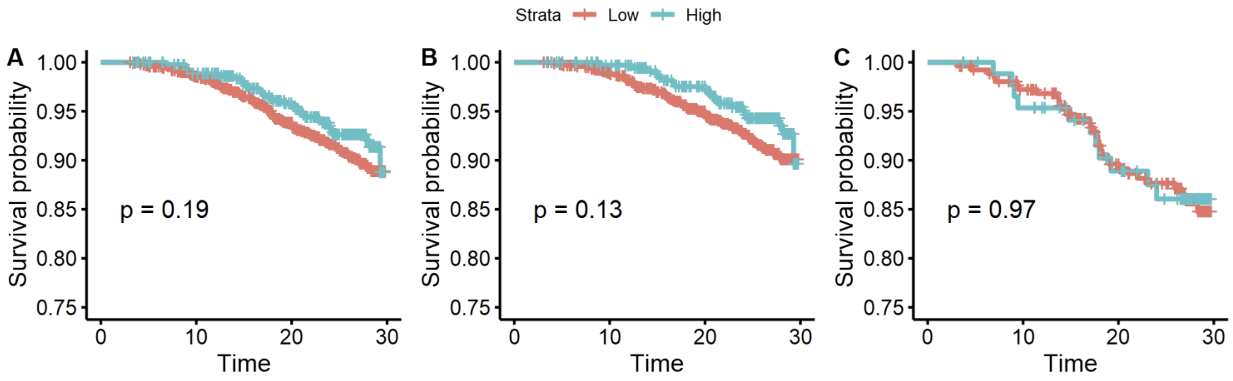

Supplemental Figure S3. Kaplan-Meier Plot Illustrating the Survival Analysis for Alzheimer's Disease (AD) in Relation to PCSK9 Protein Levels

This Kaplan-Meier plot (KM plot) visually depicts the survival analysis conducted for Alzheimer's Disease (AD) with respect to the levels of PCSK9 protein. PCSK9 Protein Levels were grouped in two low vs high use the median as the cut-off value. The analysis utilizes PCSK9 protein levels as a potential predictive factor for the occurrence of AD. Each line in the plot corresponds to a different level or category of PCSK9 protein, demonstrating the cumulative proportion of individuals without AD over time.

Panel A: All subjects, Panel B: APOE ε4 non-carriers, Panel C: APOE ε4 carriers

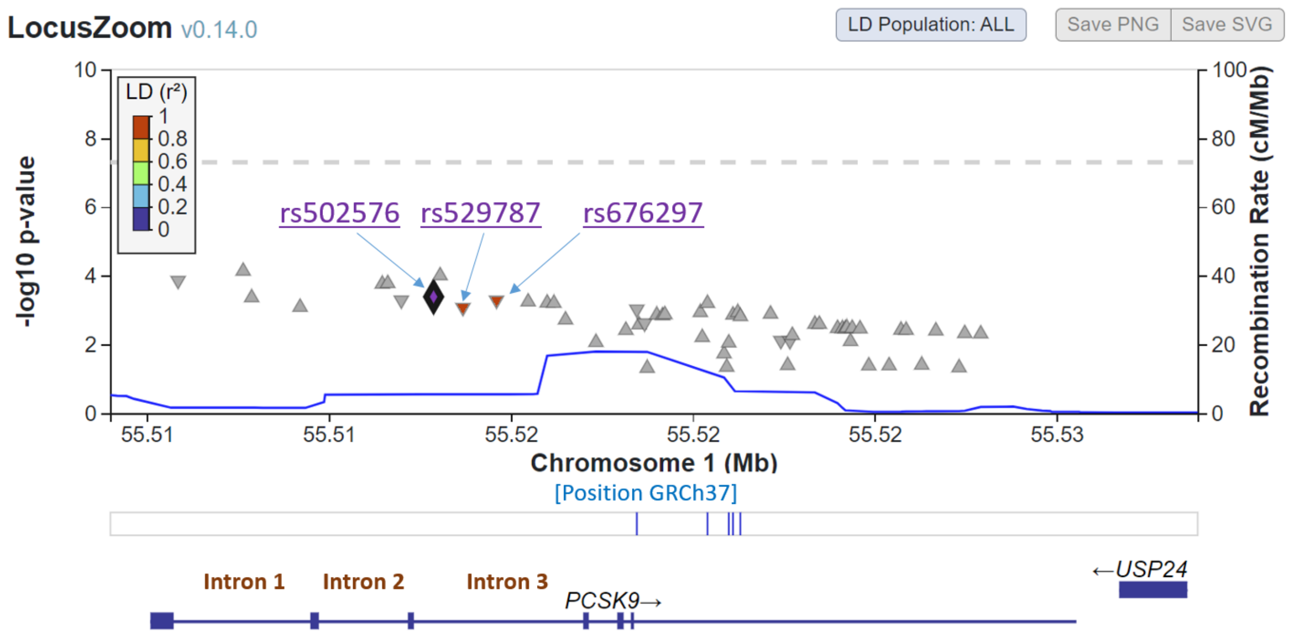

Chr	rsid	Position GRCh37	Position GRCh38	Noneffect Allele	Effect Allele	iMAF	Beta	SE	P value	Ref. gene	Genotypes
1	rs502576	55512882	55042209	C	G	0.1813	0.22	0.06	0.00041	PCSK9	0=CC, 1 = CG, 2 = GG
1	rs529787	55513521	55047209	G	C	0.1767	0.21	0.06	0.00085	PCSK9	0=GG, 1 = CG, 2 = CC
1	rs676297	55514611	55048938	T	A	0.1810	0.22	0.06	0.00052	PCSK9	0=TT, 1 = AT, 2 = AA

Supplemental Figure S4. The LocusZoom plot of the three selected SNPs and their association with PCSK9 protein levels. A linear regression model was used to assess the association between z-score-transformed PCSK9 protein levels and genetic variation, adjusting for sex, age, and the first 10 principal components (PCs) to account for population structure in the GWAS analysis.

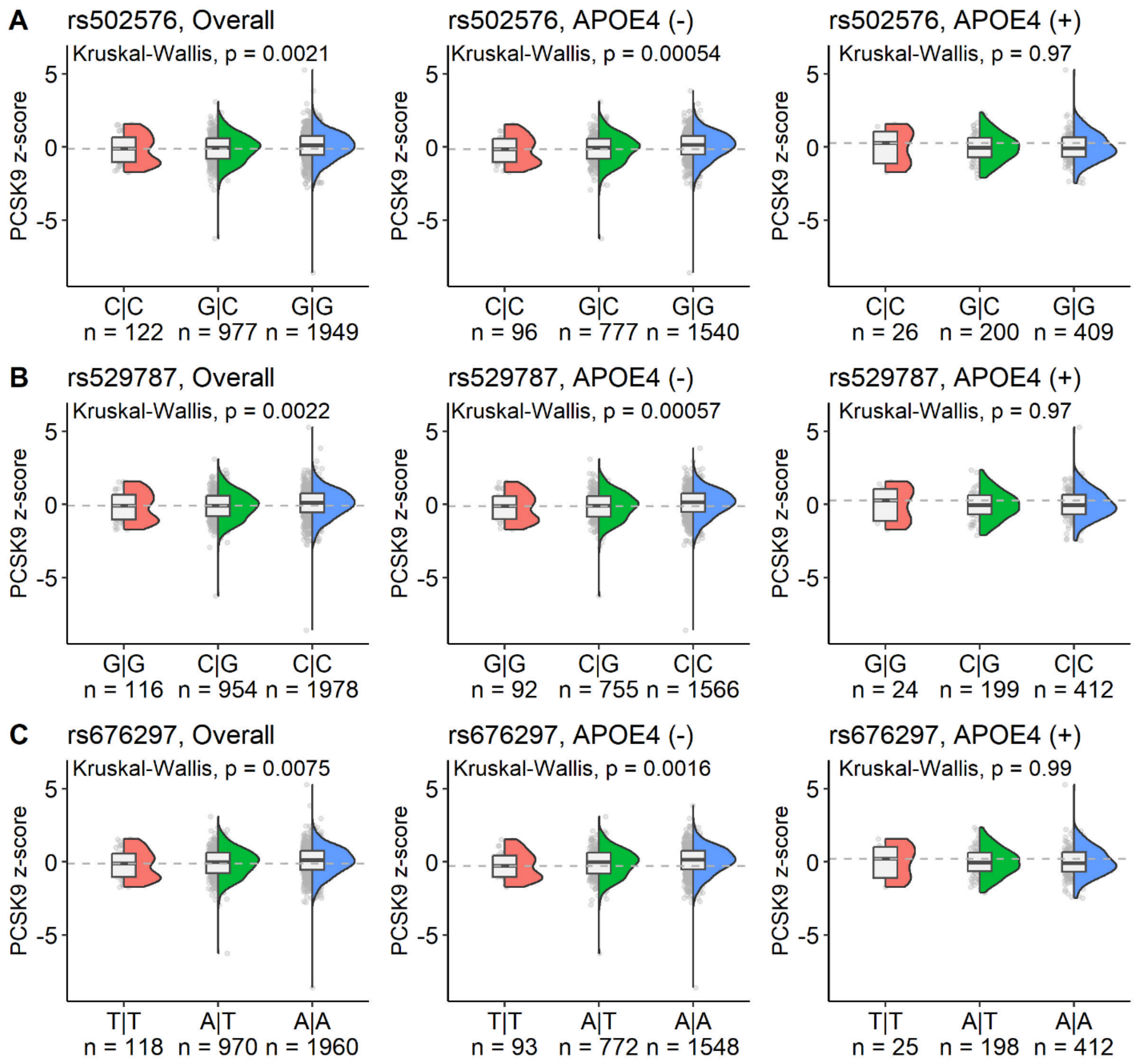

Supplemental Figure S5. **The boxplots of the blood PCSK9 protein levels across three PCSK9 genotypes in the absence and the presence of APOE ε4 allele**

Box plots for the Z-scores of the blood PCSK9 protein concentrations in FHS were illustrated based on the genotypes of PCSK9 gene (A), and then further stratified by the absence (B) and the presence (C) of APOE ε4 allele. Three genotypes for rs502576, as CC (Low), GC (Middle), and GG (High); for rs529787 were labeled as GG (Low), CG (Middle), and GG (High); and for rs676297, as TT (Low), AT (Middle), and AA (High) are shown. The labels "Low", "Middle", and "High" are referred to the corresponding PCSK protein levels in the blood in the total sample (A). The p values were from the Kruskal-Wallis tests to compare the PCSK9 levels across the three genotypes of each one of the three SNPs.

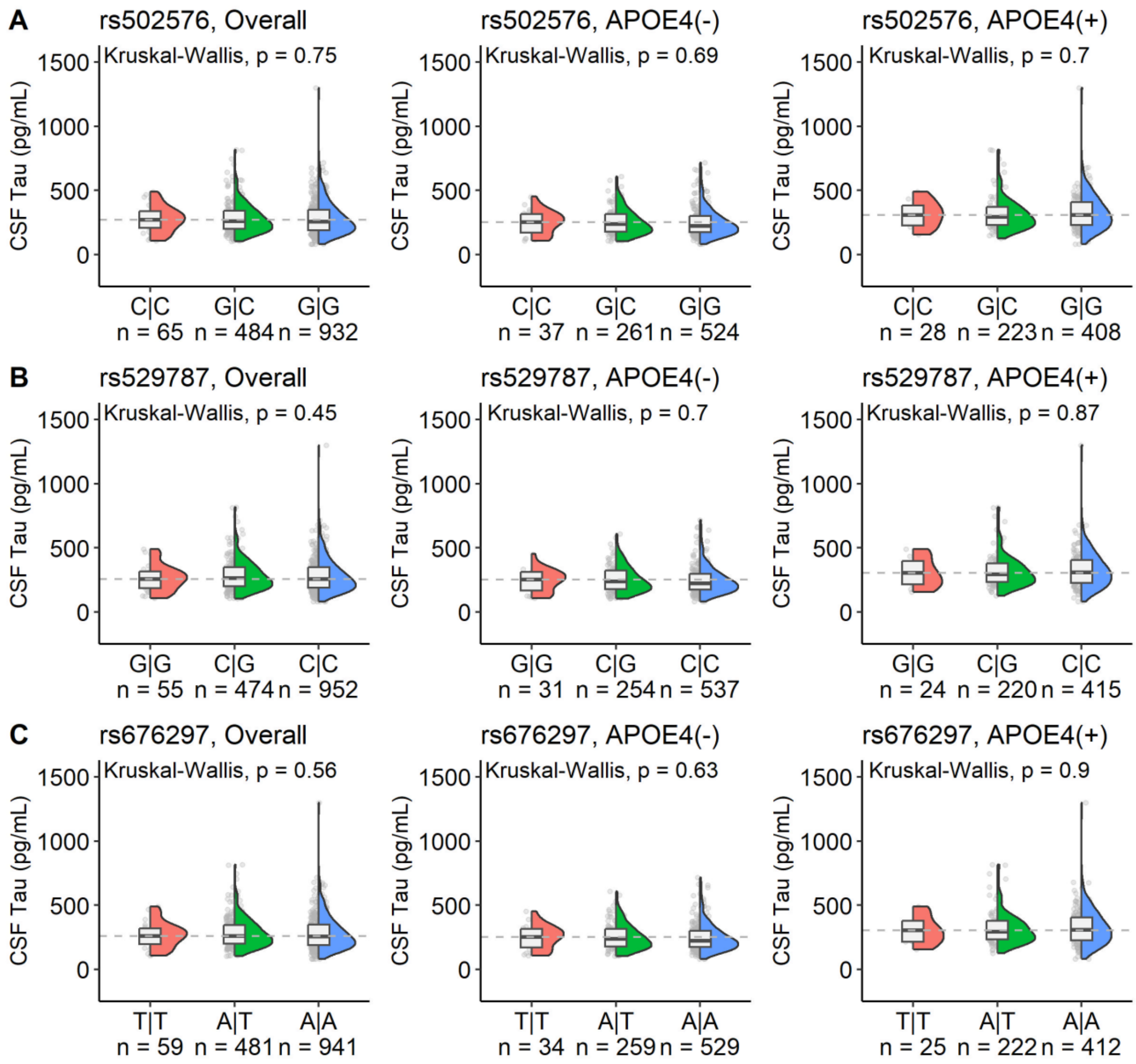

Supplemental Figure S6. The boxplots of the CSF total Tau levels among different genotypes of the three PCSK9 SNPs in the ADNI study at the baseline exams, stratified by APOE ε4 carriers' status.

The genotypes of the three SNPs were categorized from low to high based on their association with blood PCSK9 protein levels in the FHS study, as PCSK9 protein measurements were not available in the ADNI study. The label "Low", "Middle", and "High" of each genotypes were based on the PCSK protein levels in the APOE ε4 non-carriers group as shown in Supplemental Figure S5, respectively. The p values were from the Kruskal-Wallis tests to compare the CSF Tau levels across the three genotypes of each one of the three SNPs.

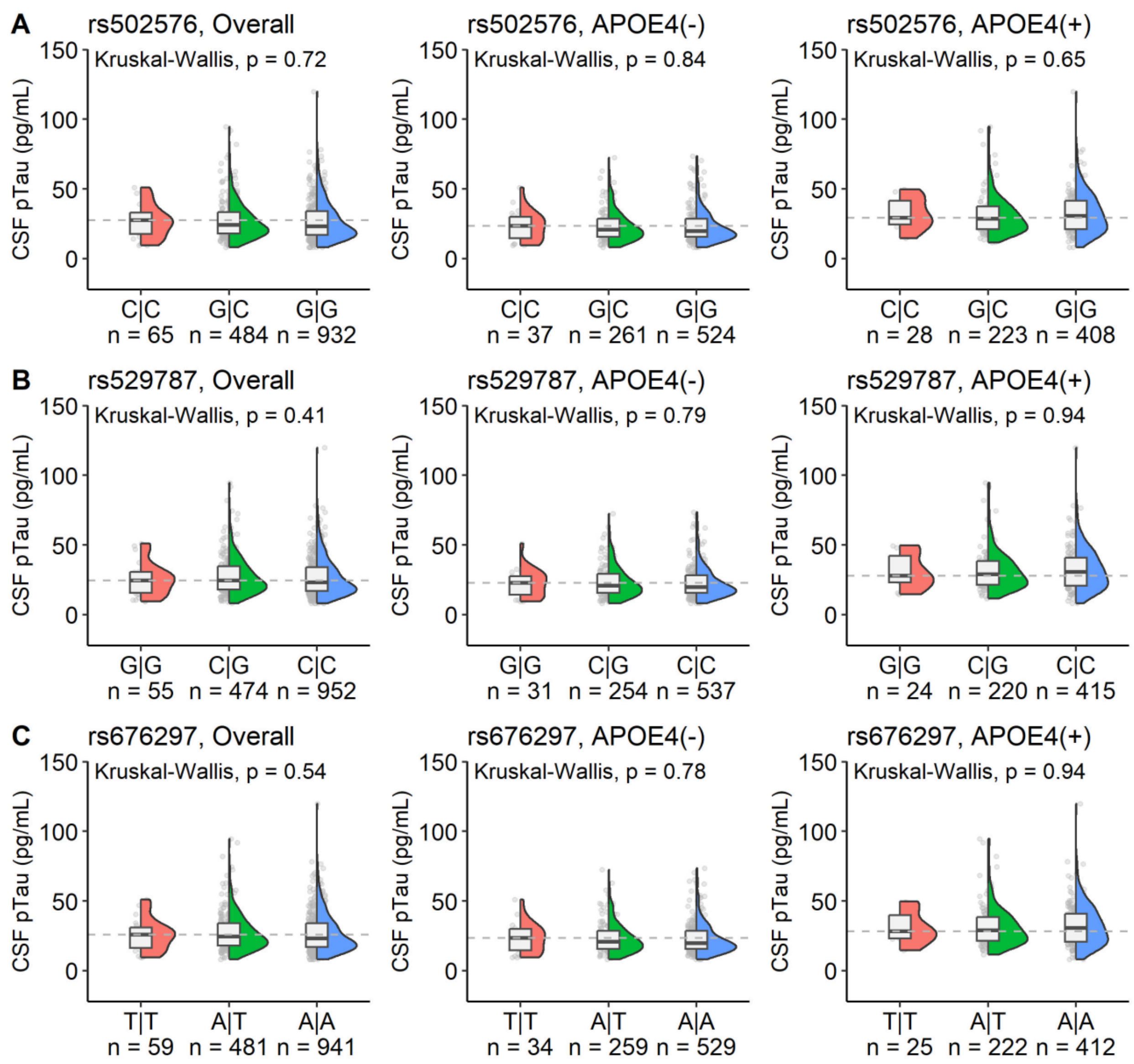

Supplemental Figure S7. The boxplot shows the CSF pTau levels among different genotypes of the three PCSK9 SNPs in the ADNI study at the baseline exams, stratified by APOE ε4 carriers' status.

The genotypes of the three SNPs were categorized from low to high based on their association with blood PCSK9 protein levels in the FHS study, as PCSK9 protein measurements were not available in the ADNI study. The label “Low”, “Middle”, and “High” of each genotypes were based on the PCSK protein levels in the APOE ε4 non-carriers group as shown in Supplemental Figure S5, respectively. The p values were from the Kruskal-Wallis tests to compare the CSF pTau levels across the three genotypes of each one of the three SNPs.

Supplemental Table S1. Baseline characteristics of two FHS datasets: the protein dataset and the genetic dataset

Baseline characteristics ** Mean ± SD or n (%)	Protein dataset § N = 1704	Genetic dataset ‡ N = 3048
**Age, years**	54.7 ± 9.88	54.5 ± 9.80
**Sex, Female**	913 (53.6%)	1617 (53.1%)
**Education**		
High school did not graduate	78 (4.6%)	156 (5.1%)
High school graduate	523 (30.7%)	933 (30.6%)
Some College	488 (28.6%)	865 (28.4%)
College graduate	593 (34.8%)	1054 (34.6%)
**APOE, ε4 carriers †**	361 (21.2%)	635 (20.8%)
**Cardiovascular diseases (CVD) #**	102 (6.0%)	266 (8.7%)
**Body mass index (BMI), kg/m^2**	27.5 ± 5.0	27.4 ± 4.9
**Cumulative use of anti-lipid medication (%) ***		
mean ± SD	2.67 ± 10.04	2.61 ± 9.69
Median (range)	0 (0, 100%)	0 (0, 100%)
**Lipid profile of the blood test**		
Total cholesterol (TC), mg/dL	205.2 ± 36.5	205.0 ± 37.1
Triglycerides (TG), mg/dL	147.7 ± 105.5	147.1 ± 115.1
HDL-Cholesterol (HDL-C), mg/dL	50.2 ± 15.0	50.1 ± 15.0
LDL-Cholesterol (LDL-C) , mg/dL ♯	126.6 ± 32.7	126.7 ± 33.0

** We used the fifth wave of the core exam (exam 5) of FHS offspring cohort as the baseline, when the blood PCSK9 level were measured.

† APOE ε4 carrier's status: APOE4 (-) were ε4 non-carriers (included three genotypes: 22, 23, 33), and APOE4 (+) were ε4 carriers (included two genotypes: 34, 44), APOE genotype 24 were excluded from this study.

\# Cardiovascular diseases (CVDs) defined as incident coronary heart disease, stroke, heart failure, and peripheral arterial disease.

* Cumulative percentage of time on anti-lipids medication was calculated to reflect cumulative exposure to anti-lipids treatment ([# of exams with medication use/# of attended observation examination] × 100).

♯ The Friedewald formula (FF) is an estimation of LDL-c level that uses the following levels of total cholesterol (TC), triglycerides (TG), and high-density lipoprotein cholesterol (HDL-c):
LDL-c (mg/dL) = TC (mg/dL) − HDL-c (mg/dL) − TG (mg/dL)/5.

§ The missing data from the protein dataset included: education = 22 (1.3%), BMI = 3 (0.2%), TC = 0 (0%), TG = 0 (0%), HDL = 5 (0.3%), and LDL = 50 (2.9%), respectively.

‡ The missing data from the genetic dataset included: education = 40 (1.3%), BMI = 12 (0.4%), TC = 12 (0.4%), TG = 12 (0.4%), HDL = 21 (0.7%), and LDL = 92 (3.0%), respectively.

Supplemental Table S2. The association between PCSK9 protein level and the dosage of PCSK9 SNPs

RSID	Chromosome	Location GRCh38	noneffect	effect	iMAF	Beta	se	p value	inrefgene	refgenes_60kb_from_SNP
rs502576	1	55512882	C	G	0.1813	0.22	0.06	0.00041	PCSK9	TMEM61;BSND;PCSK9;USP24
rs676297	1	55514611	T	A	0.1810	0.22	0.06	0.00052	PCSK9	TMEM61;BSND;PCSK9;USP24
rs529787	1	55513521	G	C	0.1767	0.21	0.06	0.00085	PCSK9	TMEM61;BSND;PCSK9;USP24
rs2479410	1	55505861	G	A	0.3437	-0.19	0.05	0.00014	PCSK9	TMEM61;BSND;PCSK9;USP24
rs6681159	1	55507882	C	T	0.7555	0.19	0.05	0.00043	PCSK9	TMEM61;BSND;PCSK9;USP24
rs10888897	1	55513061	T	C	0.5976	0.19	0.05	0.00010	PCSK9	TMEM61;BSND;PCSK9;USP24
rs11206513	1	55507649	C	T	0.5924	0.18	0.05	0.00007	PCSK9	TMEM61;BSND;PCSK9;USP24
rs10888896	1	55509213	G	C	0.7669	0.18	0.05	0.00082	PCSK9	TMEM61;BSND;PCSK9;USP24
rs7530425	1	55511471	C	T	0.5976	0.18	0.05	0.00017	PCSK9	TMEM61;BSND;PCSK9;USP24
rs11436234	1	55511623	T	TC	0.5959	0.18	0.05	0.00017	PCSK9	TMEM61;BSND;PCSK9;USP24
rs644000	1	55511995	A	G	0.3434	-0.17	0.05	0.00052	PCSK9	TMEM61;BSND;PCSK9;USP24
rs7543163	1	55515481	C	T	0.6083	0.17	0.05	0.00057	PCSK9	TMEM61;BSND;PCSK9;USP24
rs11206514	1	55516004	C	A	0.6064	0.17	0.05	0.00062	PCSK9	TMEM61;BSND;PCSK9;USP24
rs11206515	1	55516188	C	T	0.6090	0.17	0.05	0.00063	PCSK9	TMEM61;BSND;PCSK9;USP24
rs10888898	1	55516508	A	G	0.6287	0.16	0.05	0.00192	PCSK9	TMEM61;BSND;PCSK9;USP24
rs2495477	1	55518467	A	G	0.3969	-0.14	0.04	0.00095	PCSK9	BSND;PCSK9;USP24
rs2479413	1	55518682	C	T	0.3498	-0.14	0.05	0.00239	PCSK9	BSND;PCSK9;USP24
rs572512	1	55517344	C	T	0.3938	0.13	0.05	0.00866	PCSK9	TMEM61;BSND;PCSK9;USP24
rs625619	1	55518166	G	A	0.5843	0.13	0.05	0.00386	PCSK9	BSND;PCSK9;USP24
rs494198	1	55518528	C	A	0.6448	0.13	0.04	0.00266	PCSK9	BSND;PCSK9;USP24

The linear model using the z-score of the log10 transformed blood PSCK9 protein level and top SNPs of with iMAF >5%, all models control for sex, age, and 10 Principal Components (PCs) related to population structure, cryptic relatedness, and batch effects. The table ordered by the absolute value of Beta value and only the top 20 SNPs of the 56 filtered SNPs of the PCSK9 genes were shown.

Supplemental Table S3. The numbers of study subjects in the stratification of PCSK9 genotypes in the absence and the presence of APOE ε4 genotype

PCSK9 SNP genotype	APOE ε4 non-carrier (22, 23, 33) n = 2413	APOE ε4 carrier		Overall n = 3048
		heterozygous (34) n = 582	homozygous (44) n = 53	
**rs502576**				
**C|C: low**	96 (79%)	24 (20%)	2 (2%)	122
**G|C: middle**	777 (80%)	182 (19%)	18 (2%)	977
**G|G: high**	1540 (79%)	376 (19%)	33 (2%)	1949
**rs529787**				
**G|G: low**	92 (79%)	22 (19%)	2 (2%)	116
**C|G: middle**	755 (79%)	183 (19%)	16 (2%)	954
**C|C: high**	1566 (79%)	377 (19%)	35 (2%)	1978
**rs676297**				
**T|T: low**	93 (79%)	23 (19%)	2 (2%)	118
**A|T: middle**	772 (80%)	183 (19%)	15 (2%)	970
**A|A: high**	1548 (79%)	376 (19%)	36 (2%)	1960

Supplemental Table S4. Stratification and logistic regression analyses for the association between PCSK9 genotypes and the AD or all-cause dementia in ADNI study in the absence and the presence of APOE ε4 genotype

Strata	SNP	Genotype	Alzheimer's disease				All-cause dementia			
			n	events	OR [95CI]	p value	n	events	OR [95CI]	p value
**APOE4 (-)**	**rs502576**	CC: Low	37	7			37	7		
		GC: Middle	261	28	0.52 [0.20, 1.33]	0.17	261	28	0.52 [0.20, 1.32]	0.17
		GG: High	522	40	0.33 [0.13, 0.82]	0.02	524	42	0.35 [0.14, 0.86]	0.02
	**rs529787**	GG: Low	31	6	ref	-	31	6	ref	-
		CG: Middle	254	29	0.47 [0.17, 1.27]	0.13	254	29	0.47 [0.17, 1.27]	0.14
		CC: High	535	40	0.27 [0.10, 0.72]	0.009	537	42	0.29 [0.11, 0.76]	0.01
	**rs676297**	TT: Low	34	6			34	6		
		AT: Middle	259	29	0.55 [0.21, 1.50]	0.24	259	29	0.55 [0.21, 1.49]	0.24
		AA: High	527	40	0.33 [0.13, 0.88]	0.03	529	42	0.35 [0.13, 0.92]	0.03
**APOE4 (+)**	**rs502576**	CC: Low	28	7			28	7		
		GC: Middle	219	47	0.70 [0.28, 1.79]	0.46	223	50	0.75 [0.30, 1.90]	0.54
		GG: High	408	94	0.78 [0.32, 1.94]	0.60	408	94	0.79 [0.32, 1.95]	0.61
	**rs529787**	GG: Low	24	7	ref	-	24	7	ref	-
		CG: Middle	218	45	0.53 [0.20, 1.39]	0.33	220	46	0.55 [0.21, 1.43	0.22
		CC: High	413	96	0.63 [0.25, 1.60]	0.22	415	98	0.65 [0.26, 1.64]	0.36
	**rs676297**	TT: Low	25	7			25	7		
		AT: Middle	220	46	0.57 [0.22, 1.47]	0.24	222	47	0.58 [0.23, 1.50]	0.26
		AA: High	410	95	0.66 [0.26, 1.65]	0.37	412	97	0.67 [0.27, 1.69]	0.40

ADNI participants were classified by APOE ε4 status: APOE4 (-) (ε22, ε23, ε33) and APOE4 (+) (ε34, ε44), excluding ε24 carriers. Genotypes were categorized as rs502576 (CC-Low, GC-Middle, GG-High), rs529787 (GG-Low, CG-Middle, GG-High), and rs676297 (TT-Low, AT-Middle, AA-High). The label "Low", "Middle", and "High" of each genotypes were based on the PCSK protein levels in the APOE ε4 non-carriers group as shown in the Supplemental Figure S5, respectively. Using the lowest PCSK9 genotype as reference, logistic regression estimated odds ratios (OR) and 95% confidence intervals (CI) for AD or all-cause dementia within each APOE group.
